# A Multi-Context Regulome-Wide Association Atlas for Genetic Studies of Aging Brain Disorders

**DOI:** 10.64898/2026.05.15.26353329

**Published:** 2026-05-19

**Authors:** Chunming Liu, Anqi Wang, Hao Sun, Kaixuan Luo, Sheng Qian, Yining Li, Xin He, Philip De Jager, David Bennett, Minghui Wang, Carlos Cruchaga, The Alzheimer’s Disease Functional Genomics Consortium, Gao Wang, Fabio Morgante

## Abstract

Genome-wide association studies have identified risk loci for aging brain disorders, but mechanistic interpretation depends on linking these loci to genes and to the tissues, cell types, and molecular modalities in which those genes act. Here we introduce FunGen-xQTL Multi-Brain (FGMB), a multi-context regulome-wide association atlas for transcriptome-wide association studies (TWAS) built from molecular datasets assembled by the ADSP Functional Genomics Consortium. FGMB provides *cis* -genetic prediction models for 17,375 protein-coding genes across 36 molecular datasets, 18 contexts, and 3 regulatory modalities, yielding more than 293,000 imputable gene-level or splice-event models. FGMB evaluates eight established and newer Bayesian or multivariate prediction methods, including cross-context models that borrow information across tissues and cell types. Applied to Alzheimer’s disease, FGMB identified 327 TWAS associations and used joint fine-mapping of variants and predicted molecular traits to prioritize 146 gene–molecular-trait pairs, distinguishing regulatory associations from linkage disequilibrium (LD) hitchhiking.

## Full Text Availability

The license terms selected by the author(s) for this preprint version do not permit archiving in PMC. The full text is available from the preprint server.

